# Effect of the AWJM Method on the Machined Surface Layer of AZ91D Magnesium Alloy and Simulation of Roughness Parameters Using Neural Networks

**DOI:** 10.3390/ma11112111

**Published:** 2018-10-26

**Authors:** Ireneusz Zagórski, Mariusz Kłonica, Monika Kulisz, Katarzyna Łoza

**Affiliations:** 1 Department of Production Engineering, Mechanical Engineering Faculty, Lublin University of Technology, 20-618 Lublin, Poland; m.klonica@pollub.pl (M.K.); katarzyna.loza@pollub.edu.pl (K.L.); 2 Department of Enterprise Organisation, Management Faculty, Lublin University of Technology, 20-618 Lublin, Poland; m.kulisz@pollub.pl

**Keywords:** AWJM, cutting effectiveness, magnesium alloys, surface quality, artificial neutral networks

## Abstract

This paper investigates the effect of change of the abrasive flow rate and the jet feed on the effectiveness of machining of AZ91D casting magnesium alloy. The evaluation of the state of the workpiece surface was based on surface and area roughness parameters (2D and 3D), which provided data on: irregularities formed on the workpiece edge surface (water jet exit), the surface quality after cutting, the workpiece surface chamfering, microhardness of the machined surface, and of specimen cross-sections (along the water jet impact). The process was tested for two parameter settings: abrasive flow rate 50 at cutting speed v_f_ = 5–140 mm/min, and abrasive flow rate 100% (0.5 kg/min) at v_f_ = 5–180 mm/min. The results demonstrate a significant effect of the abrasive flow rate and the jet feed velocity on the quality of machined surface (surface roughness and irregularities). In addition, selected 2D surface roughness parameters were modelled using artificial neural networks (radial basis function and multi-layered perceptron). It has been shown that neural networks are a suitable tool for prediction of surface roughness parameters in abrasive water jet machining (AWJM).

## 1. Introduction

Abrasive water jet machining (AWJM) is one of the fastest developing technological methods for cutting materials. It is used, inter alia, for effective machining of aluminium alloys and “difficult-to-machine” materials such as titanium alloys or various types of composite materials. In the case of light alloys this machining technique is approximately two times faster than in the case of steel. The machined surfaces are very smooth, so no finishing treatment is required. The AWJM process does not affect the state of the workpiece material (except for plastic deformation) due to the lack of the heat affected zone (in contrast to laser or plasma cutting), which ensures the integrity of alloy structure. Compared to drilling, the AWJM is also a more effective method for making small-diameter holes, e.g., 0.8–1.5 mm, which act as “pilot” holes. In the case of aluminium alloys, abrasive water jet cutting can be used for both thin metal sheets (e.g., with thickness lower than 3 mm) and blanks in the form of plates (blocks) with a thickness of over 150 mm. The AWJM process has the potential to become one of the most effective, unconventional subtractive machining methods (lower mechanical and thermal damage of the machined surface). However, it is necessary to study the influence of adjustable input parameters of the process, such as the water pump pressure, the material removal rate, the jet flow velocity, and the nozzle-workpiece distance, on the output quality and accuracy parameters (geometrical accuracy—straightness of the cut gap, homogeneity of the machined surface roughness measured along the direction of cut, and striation marks as well as other profile irregularities, the integrity of the machined surface—the pressing in of abrasive grains, the formation of “white” micro-layers, material pull-out, and microcracks) [1].

**Figure 1:**
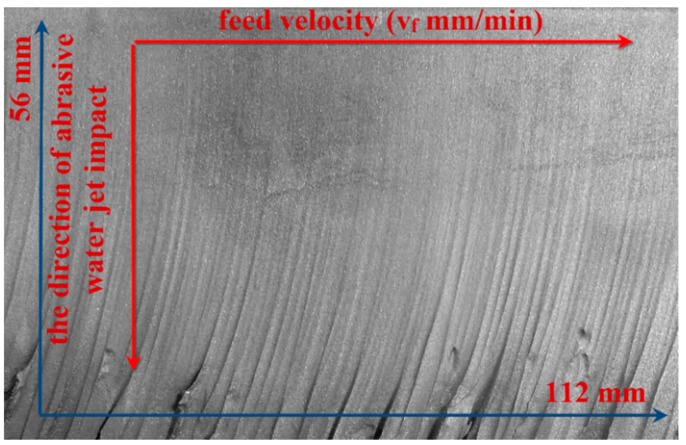
Surface of a AZ91D specimen after AWJM.

A characteristic phenomenon in high-pressure abrasive water jet cutting process is the occurrence of water jet deflection on a certain thickness of the machined material. This is due to the fact that most energy of the water jet is used to remove the upper layer of the material. On the other hand, the remaining energy is insufficient to cut the material as effectively as at the beginning of the process (on the upper edge of the specimen). With increasing the depth of water jet impact in the material, the quality of the cut deteriorates, as shown in Figure 1. This is the effect of uneven distribution of the kinetic energy of the abrasive material, which results in visible machining traces and marks on the lower surface of the cut [2], also referred to as “striation marks”. Increasing the water pressure leads to an increase in the jet feed velocity and the aerodynamic force. This shortens the compact part of the water jet and increases the tip angle of the sprayed layer. The shape of the water jet has a significant effect on the depth of cut in the material. When the jet shape is conical, increasing the distance between the nozzle and the workpiece leads to decreasing the depth of cut [3]. During the water jet cutting process, the upper edge of the workpiece is rounded while the lower edge will often show burrs, which must be removed. One of the deburring methods is brushing. The use of wire brushes allows—apart from deburring and edge rounding—the generation of the desired properties of the substrate surface layer [4].

The effectiveness of a process can be understood as the effect of machining measured by the efficiency and quality of machined surfaces or other defined quantities, obtained with specified technological parameters. The effectiveness of machining can also be defined as the ratio of incurred expenditures (in our case, predominantly the costs of tools and materials) to the value of effects obtained thanks to these expenditures. In a broader sense, the concept of process effectiveness means the best results (defined by specific quantities) obtained in the production process at the lowest costs possible. The effectiveness of manufacturing aircraft components, ensuring their high reliability, depends on a number of factors, e.g., the correct design of a product; the application of an appropriate production strategy; the use of tools with extended service life; the application of machining parameters ensuring the efficiency and at the same time high stability of the process; proper supervision of the process, and quick effective response to the loss of productivity, or the use of appropriate machine tools allowing for HSM [5,6]. On the other hand, the machined surface quality is a concept that includes surface roughness and waviness along with surface defects [7]. In practice, the quality of the machined surface is usually described with surface or area roughness parameters (2D, e.g., Ra, Rz, RSm, or 3D, e.g., Sa), and it is one of the basic absolute machinability indicators [5,8]. The influence of AWJM conditions on the material removal rate and the workpiece geometry has been widely studied. It has been found that the surface quality is to a significant extent affected by the water jet pressure, jet feed velocity, and the distance between the nozzle and the workpiece [9,10].

Borkowski [11] investigated the geometrical structure of machined surfaces of inter alia EN-AW 6082 aluminium alloy (AlSiMgMn, PA4) by analysing selected surface roughness parameters. Based on these results, it can be concluded that the geometrical structure of the machined surface largely depends on the tensile strength of the material. The experiment revealed that decreasing the water pressure leads to an increase of mean Sq. Moreover, a qualitative model of the cutting process was elaborated using the non-linear estimation module, therefore enabling the experimental approximation *via* user-indicated regression dependency. The developed model can be implemented at low costs to control machine tools for water cutting [11,12]. In Spadło [13], one of the tested substrates was EN AW-2017A alloy (AlCu4MgSi, PA6). It was observed that the jet feed velocity has a significant impact on the surface quality of the machined materials. To obtain the acceptable target surface, the jet feed velocity v_f_ should be set to 40 mm/min. A very good quality of cut is achieved by reducing the jet feed velocity from 5 to 7 times in relation to the maximum velocity used for workpiece separation. The quality of the cut area depends, among others, on the type and thickness of the material being cut (for soft composites the water pressure is set in the range of 300–400 MPa). 

In Skoczylas [14], the greatest impact of the cutting speed in the entrance zone was observed for 7175-T7351 aluminium alloy, where the change in surface roughness amounted to 30%. It was observed that the increase in the cutting speed causes an increase in surface roughness. After cutting, two zones can be distinguished on the specimen surface: the entrance zone characterized by lower surface roughness and the exit zone with machining traces, which generates higher values of the Ra parameter, as the surface roughness increases with the increasing cutting depth of the abrasive water jet. The work by Klichova [15] examines selected surface roughness parameters (Ra, Rz, Rq) depending on the feed velocity (v_f_ = 100–600 mm/min) and the strength properties of aluminium alloys. It was found that with increasing the velocity v_f_, the surface roughness parameters increase as well. The best surface quality was obtained (at the highest jet feed velocity) for EN AW-6060 alloy, whereas the worst surface quality was achieved for EN AW-7075 alloy. Zhao [16] investigated 6061 alloy with similar properties and chemical composition. At least four zones in the specimen macrostructure could be distinguished: the entrance zone, the smooth zone, the transition zone and the rough zone. Based on the morphology and microstructure examination of the machined surface, friction characteristics and machining traces were identified. The examination of specimen surface has shown that the smooth surface is obtained for materials with high hardness, while soft materials exhibit high susceptibility to erosion. Maros [17] investigated AlMgSi0.5 aluminium alloy. The formation of the so-called cone at the point of cut is one of key problems associated with water jet cutting accuracy. The cone angle increases with the increase of water jet feed velocity. The increase in pressure causes a decrease in the size of the cone angle. A similar effect can be observed when the abrasive flow rate is increased. 

Yuvaraj [18] describes the experiments of cryogenic assisted abrasive water jet (CAAWJ) cutting of AA5083-H32 aluminium alloy. The following were examined: 3D surface morphology, 2D surface topography, residual stresses, and microhardness; an X-ray powder diffraction (XRD). The work analysed varying nozzle settings and different mesh sizes of the abrasive, and concluded that the application of the CAAWJ method for aluminium alloys improves the functional properties of their surface. 

An interesting application of the investigated method is cutting out thin-walled elements from popular aluminium alloys. The maximum depth of cut often depends, among others, on the mechanical and technological properties of the workpiece. Bławucki’s study [19] presents the results of experimental tests of cutting out thin-walled elements from an aluminium alloy EN AW-2024 rod produced by rolling. The influence of technological parameters of the cutting process on the thickness of cut-out walls was analysed. It was found that there is a technical and technological possibility of cutting out thin-walled elements with a nominal wall thickness of 0.4 mm and the depth of cut 60 mm.

Ochal [20] presents selected results of the surface quality evaluation of adhesively-bonded samples after cutting. One of the analysed substrates was EN AW-2017A aluminium alloy. The specimens were adhesively bonded as classical lap joints and sandwich structures in various configurations. The tests confirmed that high-pressure abrasive water jet machining is an effective tool for cutting sandwich structures. With appropriately selected cutting parameters, high-quality machined surfaces can be obtained. 

Often, the preparation of a semi-finished product involves the machining of plates made from e.g., aluminium alloys of various thicknesses. The study [21] investigated the effect of Plain Water Jet (PWJ) machining without the use of abrasive grains from the perspective of changes in the feed velocity (up to 5000 m/min), the nozzle distance from the workpiece, the water jet pressure, and the number of passes. It was shown that the abrasive water jet generates compressive residual stresses that have a positive effect on fatigue life. Similarly, Boud [22] describes the influence of AWJC process parameters on the surface roughness (Ra) of aluminium plates. The water jet feed velocity, v_f_, was in the range of 0.75–4.2 m/s (45–252 m/min), and the modified process parameters were the following: the water jet pressure, the abrasive flow rate and the nozzle distance from the workpiece. On the other hand, the Begic-Hajdarevic work [23] investigated the effect of material thickness, feed velocity and abrasive flow rate. The experimental results showed that the water jet feed velocity has a significant effect on the surface roughness in the region of water jet exit. On the basis of the results, optimal process parameters were determined for every tested thickness of the workpiece.

Attempts are often made to predict surface roughness by means of extreme machine learning (ELM) in order to assess the roughness of a surface subjected to high-pressure abrasive water jet cutting. The results of the assessment and prediction based on the ELM model were compared with the results obtained from the genetic algorithm (GP) and artificial neural networks (ANNs). The experimental findings show that the accuracy of prediction based on the ELM model can be enhanced with the results obtained from the genetic algorithm (GP) and artificial neural networks (ANN). What is more, the developed ELM models can be effectively used in the future research on the development of innovative models and the formulation of new strategies for surface roughness prediction of workpieces machined with the abrasive water jet method [24]. Similarly, in [25] an attempt was made to predict the surface roughness parameter Ra after the AWJM process for AISI 304 stainless steel. In addition to the surface roughness analysis, phenomena such as acoustic emission and vibration were also analysed. The use of artificial neural networks also enables the prediction of other machinability indicators, such as the cutting force components, the cutting zone temperature or vibration components [26,27,28]. In addition, a set of values describing the phenomenon (factor) studied with the use of predictive models (e.g., econometric models) gives the possibility of inferring about future development of its capabilities [29].

The major advantages of the AWJM process include: the cutting operation is performed without creating a heat influx zone; no tool wear and no tool-workpiece contact; the possibility to form materials of any hardness and produce complex shapes; no dust, fumes and impurity of the workpiece. The high-pressure abrasive water jet machining technique is constantly developed to enhance both its effectiveness and the area and range of its applications. The value of water jet pressure directly affects the efficiency of the machining process. A higher flow velocity of the abrasive water jet causes faster subtraction of the material, and thus increases the efficiency of the process [1,6]. The forces occurring during machining are so small that the abrasive water jet machining process does not require the use of additional equipment [12,30].

Given the above, it seems advisable to use the AWJM technique for the pre-treatment of magnesium alloys; however, to date there have been no publications on the subject. A survey of the literature in the field has revealed that the problem undertaken in this work has not been thoroughly investigated, and the test material has not yet been applied in previous studies. In addition, magnesium alloys belong to the group of materials with limited processing properties due to the risk and danger of ignition. Therefore, for the above reasons, abrasive water jet machining can be used as a method for preparing semi-finished products (especially plates and ingots), made of light alloy substrates, for further machining operations.

## 2. Materials and Methods 

The main objective of the study was to perform an abrasive water jet machining process for AZ91D magnesium alloy specimens and to investigate the quality and effectiveness of cutting. On the one hand, the costs of the process can be reduced by decreasing the abrasive material flow rate; on the other hand, however, attempts are made to ensure the optimal effectiveness of the process. The AWJM process was executed with a modified abrasive material flow rate. In the first stage of the experiment, the pressure was set to p = 350 MPa and the abrasive material flow rate was m_a_ = 100% (0.5 kg/min), whereas in the second stage: p = 350 MPa and m_a_ = 50%. The experiments were conducted in accordance with PN-EN ISO 9013.

Table 1 shows the chemical composition of the AZ91D alloy workpiece. 

The AWJM of AZ91D magnesium alloy was performed with the use of Eckert’s Opal Waterjet Combo (Legnica, Poland) cutting machine for plasma and water cutting. The dimensions of test specimens were 112 mm × 15 mm × 56 mm. The abrasive water jet machining process was performed using GARNET 80 mesh. Compared to other abrasive media, this material is natural sand with reduced dustiness, low wear, high hardness, and good machinability due to sharp edges of garnet grains. It is one of the most widely used abrasive media. 

**Figure 2:**
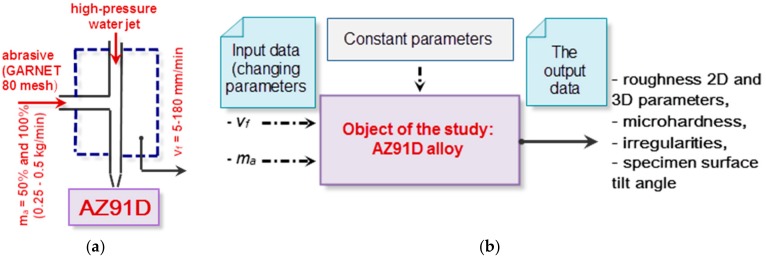
Schematic diagram (**a**) and test set-up (**b**) with the object of the study.

Table 2 gives the operational parameters of the investigated abrasive water jet machining process, whose values were determined experimentally, based on previous studies and literature survey. The schematic diagram and test plan are shown in Figure 2.

The AWJM process was followed by 2D and 3D roughness measurements of the surface produced by the abrasive water jet impact. The 2D surface roughness measurement was taken in five repetitions in two regions of the test specimen: at the water jet entrance region and in the middle of specimen height (i.e., in the middle of the semi-finished product thickness). The employed method of 2D roughness measurement enabled defining the optimal machining conditions (with respect to the target surface quality), as well as the area for 3D roughness measurements. The selected 3D area roughness parameters were examined at a 1/3 distance of specimen height from the water jet entrance region. The 2D surface roughness of test specimens was measured with Hommel’s T1000 roughness tester. Technological parameters for the 2D surface roughness measurement were as follows: ISO 11562 filter (M1), sampling length, Lc = 0.8 mm; measuring length, Lt = 4.8 mm; traverse feed velocity, v_t_ = 0.5 mm/s. 3D surface roughness and topography measurements were made with the T8000RC120-400 profilographometer by Hommel-Etamic Jenooptik (Jena, Germany). The results obtained from the measurements were processed by means of Hommel Map. The irregularities on the specimen surface were examined with the VHX 5000 digital microscope from Keyence (Osaka, Japan), equipped with VH-2 100R optics. The notion of irregularity pertains to the spacing between the highest peak and the deepest valley created due to the water jet impact on the specimen surface. The specimen surface tilt angle after machining was measured with the Vista ZEISS ( Jena, Germany) coordinate measuring machine. These measurements were made along the direction of the abrasive water jet cut. The measurements were carried out with the RENISHAW PH10M probe head with TP20 touch-trigger probe kit and Renishaw A-5003-0047 stylus, of the following technical specification: ball diameter d = 5 mm; effective working length l = 30 mm; ball weight of 2.57 g. The results of the specimen tilt angle measurements (specimen chamfering) were processed in Power Inspect 4.3.5.1. The measurements were made in the touch and learn mode. Subsequently, the measuring cycle was saved and the measurement of successive specimens was done automatically. 

In addition, the effect of machining parameters on the machined surface microhardness was measured by means of the Leco LM700AT (St. Joseph, MI, USA) microhardness tester with a diamond pyramid-shaped indenter with a square base and the tilt angle of the opposite specimen walls 136° (Vickers method). The indenter load was set to 10 g. This microhardness tester has a resolution of 0.1 HV. The specimen microhardness was tested for two variants: first, we examined the microhardness of the specimen surface, then on the specimen lateral surface, along the jet impact direction. The experiments were performed in compliance with the standard PN-EN ISO 6507-1:2018-05.

**Figure 3:**
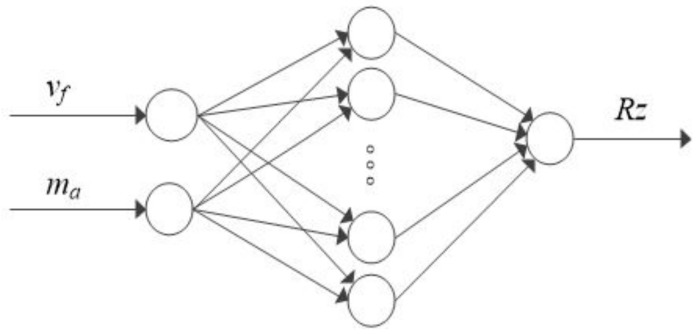
Schematic representation of an artificial neural network for the roughness parameter Rz.

In addition, to predict the nonlinear technological process, i.e., abrasive water jet machining, selected 2D surface roughness parameters (Ra, Rz, RSm) were simulated. The simulations were performed in Statistica software using artificial neural networks and their results may be used to create a decision process support system. Two types of networks were employed: radial basis function (RBF) and multi-layered perceptron (MLP). The training of the MLP was done by the BFGS (Broyden-Fletcher-Goldfarb-Shanno) method. The MLP modelling was performed using the following activation functions: linear, exponential, logistic, tanh, and sinusoidal. The activation function of the RBG for hidden neurons is the Gaussian distribution and for the output neurons—a linear function. The most important indicators of network fitting were: training quality, validation quality, and training error determined by the method of least squares. The simulations were performed for three different surface roughness parameters, which yielded three models. An example of the simulation is shown in Figure 3, surface roughness parameters are produced at the model output. In the simulations, the emphasis was placed on obtaining the simplest network structure possible, therefore, all networks have one hidden layer. The input layer consists of two neurons (the jet feed velocity v_f_ and the abrasive flow rate m_a_), while the output layer contains one neuron (a surface roughness parameter, Ra, Rz and Rsm, respectively). The number of training epochs was 200 and the hidden neurons (1–10) were selected experimentally. 18 sets of machining parameters were used for network training. The training dataset comprised of 75% measurement results while the validation dataset—25%. Due to a small number of datasets, the test dataset was omitted [31].

## 3. Results and Discussion

### 3.1. Microscopic Examination of Machined Specimen Surface 

**Figure 4:**
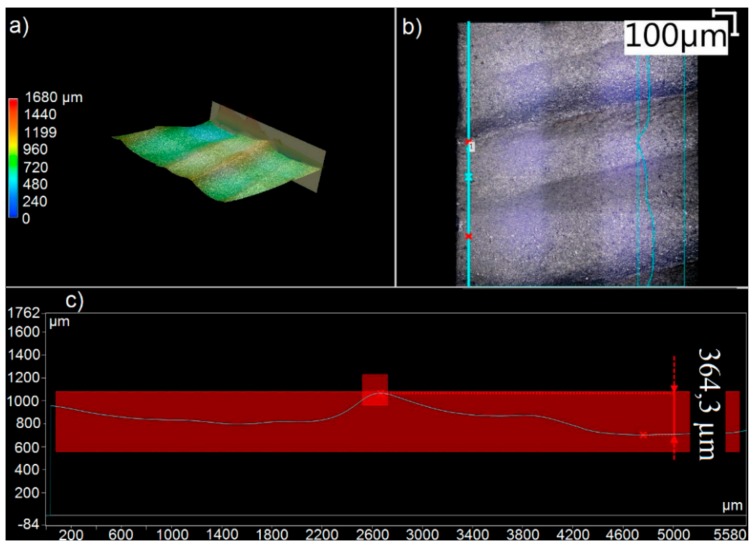
Example of a microscopic examination report of the measurement of irregularities in the jet exit region at lower specimen edge (m_a_ = 50%, v_f_ = 80 mm/min): (**a**) 2D view, (**b**) 3D view, and (**c**) typical surface irregularity measurement.

The diagrams show the results of microscopic examination—the measurements of the irregularities, i.e., the spacing between the highest peak and the deepest valley in the tested region of the lower, rough surface of the specimen. Figure 4 shows an example of the measurement of irregularities made in the jet exit region at the lower edge of the specimen. 

**Figure 5:**
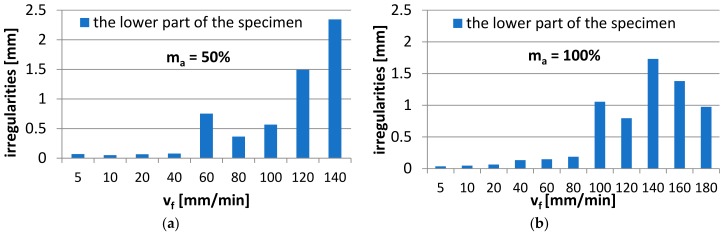
Irregularities in the examined region of the lower part of the specimen for the abrasive material flow rate: (**a**) 50% and (**b**) 100%.

Figure 5a,b show the numerical values of irregularities obtained from microscopic examination reports for the jet exit region, at the lower edge of the specimen after machining. In addition, a trend line approximated with a power function is shown. 

The diagrams in Figure 5a,b reveal that at the jet feed velocity v_f_ = 40 mm/min, the quality of cut surface is acceptable due to low irregularity values—below 0.1 mm for m_a_ = 50%, while for m_a_ = 100% —it is below 0.1 mm for the range of v_f_ = 5–20 mm/min and below 0.2 mm for the range of v_f_ = 40–80 mm/min, respectively. This means that the specimens produced at the above ranges of v_f_ do not have to undergo finish machining at a later stage of the process. The elimination of finishing results in a shorter pre-treatment as well as reduced production costs already at the stage of semi-finished product preparation. The highest irregularity occurs for the maximum jet feed velocity and the abrasive material flow rate of 50%, and is approx. 2.5 mm. It was also observed that the irregularities are almost two times higher for the abrasive material flow rate of 50% and higher jet feed velocities than those observed when the flow rate was 100%. 

### 3.2. Specimen Surface Chamfering

**Figure 6:**
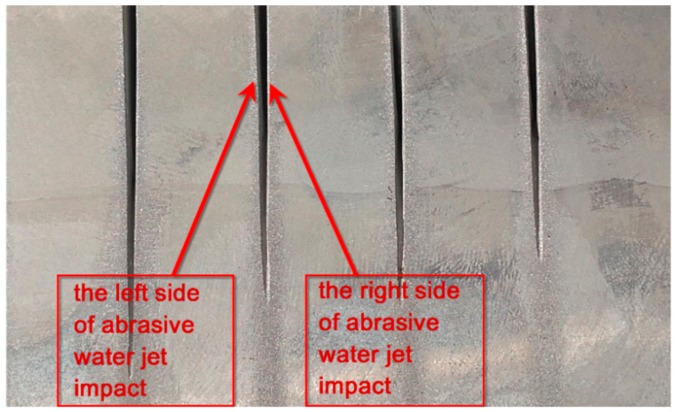
Effect of water jet action, the creation of “jet impact sides”.

**Figure 7:**
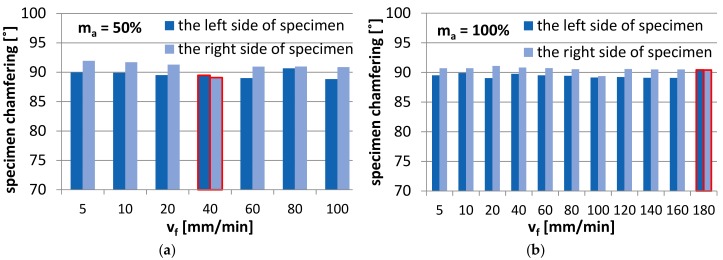
Comparison of specimen chamfering after AWJM depending on the jet impact side, for the abrasive flow rate: (**a**) 50%, and (**b**) 100%.

During the process, the specimen was subjected to impact of the abrasive water jet from left and right side of the specimen. As a result, every cut produced two surfaces cut with the same jet feed velocity. Figure 6 shows the sides of abrasive water jet impact on the specimen surface, whereas Figure 7a,b show the results of specimen chamfering after the abrasive water jet machining process depending on the side of water jet impact.

The results obtained for the abrasive material flow rate of 100% are shown for the jet feed velocities of up to v_f_ = 180 mm/min, while for the abrasive material flow rate of 50% for the jet feed velocity of up to v_f_ = 100 mm/min. The differentiation between the two jet feed velocity ranges results in the presence of high irregularities on the machined surface and the lack of full cut of the specimen at extreme values of the jet feed velocity applied in the experiment. Due to high irregularities on the specimen surface, it was impossible to perform the measurement for m_a_ = 50% and v_f_ = 120–140 mm/min (measuring programme error). 

The diagrams reveal that the specimen surfaces subjected to AWJM undergo chamfering. The tilt angle of the specimen surface on the left side of jet impact is smaller than 90°. In contrast, the tilt angle of the specimen on the right side of jet impact is higher than 90°. Nonetheless, there are two exceptions from this dependence:

- when the abrasive material flow rate is 100% and v_f_ = 180 mm/min, the tilt angle on the right and left sides of jet impact is the same,
- when the abrasive material flow rate is 50% and v_f_ = 40 mm/min, the tilt angle on the left and right sides of jet impact is smaller than 90°.

The slight differences in the specimen chamfer angle are practically insignificant from a technological point of view. However, they could be analysed statistically. For the least favourable case, the absolute tilt angle was 1.94° for the abrasive flow rate of 50% and 1.09° for the flow rate of 100%. To make the specimen surface orthogonal would require employing finish machining. If a smaller thickness of the machined elements were specified, the effect of specimen lateral surface chamfering could probably be omitted. 

### 3.3. Surface Roughness 

**Figure 8:**
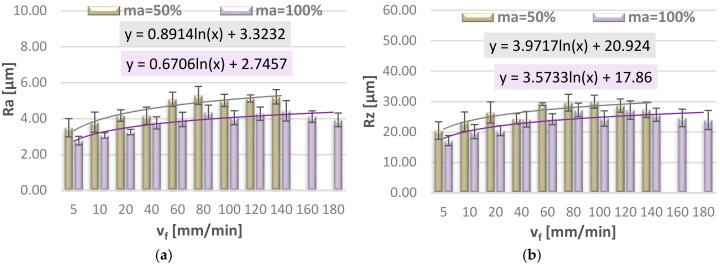
Jet feed velocity v_f_ versus surface roughness parameters: (**a**) Ra and (**b**) Rz.

**Figure 9:**
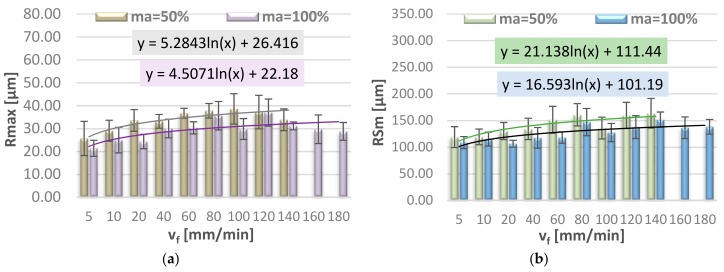
Jet feed velocity v_f_ versus surface roughness parameters: (**a**) Rmax and (**b**) RSm.

Figure 8 and Figure 9 show the effect of jet feed velocity on selected 2D surface roughness parameters (Ra, Rz, Rmax, RSm), measured on the lateral surface of the specimen, in the region of water jet entrance. In order to provide interpretation of the obtained results we can draw a function representing the trend—the logarithmic function with the description of the equation. The logarithmic function provides the best fit of the approximation curve to the mean values of surface roughness parameters.

In the jet entrance region one can observe that all analysed 2D surface roughness parameters have a tendency to increase up to approx. 80–100 mm/min, after that they become stable and then the values of Ra, Rz, Rmax, and RSm decrease. As for the parameters measured in the middle of the specimen height, it is observed that the mean values of Ra and Rz first increase to the jet feed velocity of v_f_ = 20 mm/min and then become stable. 

Higher values of surface roughness parameter Rz may exert a negative impact on the operating and strength characteristics of the machine parts, such as material strength, intensification of friction and wear processes, or reduction of mechanical tightness properties. It is, therefore, of considerable importance that the cutting velocity should be suitable for the given application.

Table 3 lists a selection of the isometric images of AZ91D specimen surfaces after abrasive water jet machining. Letter A denotes the abrasive water jet entrance while B marks its exit. In the isometric images of specimen surface one can clearly notice deflected striation marks in the exit region of the abrasive water jet. The striation marks are much clearer for the specimens cut with the jet feed velocities higher than 60 mm/min. The region of the water jet entrance is characterized by a uniform structure with lower surface roughness parameters, compared to the exit region which has a more varied structure.

**Figure 10:**
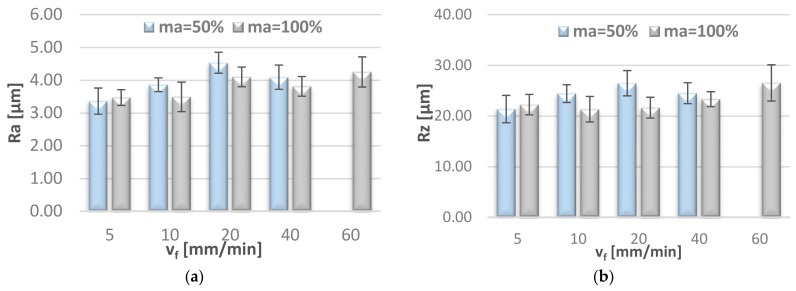
Effect of jet feed velocity v_f_ on surface roughness parameters: (**a**) Ra and (**b**) Rz, measured in the middle of specimen height.

Figure 10 illustrates the effect of jet feed velocity on selected 2D surface roughness parameters (Ra, Rz), measured on the lateral surface of the specimen, in the middle of specimen height.

Table 4 gives selected abrasive material curves resulting from specimen machining. The table shows the abrasive material curves for three jet feed velocities: (5, 60 and 140) mm/min. The analysed surface roughness parameters were measured along X axis (scallop in longitudinal feed), while along the Y axis the traverse feed was performed (the number of scallop lines) along the direction of the abrasive water jet cut.

From an operational point of view, the abrasive material curve provides vital information about a given surface [32]. It is known that the curve can be of varied nature, digressive, progressive, progressive/digressive, or digressive/progressive. In terms of operational characteristics of a surface, the most favourable curves are of progressive or progressive-digressive nature. Based on the obtained results the curves given in Table 4, it can be stated that the surfaces of AZ91D specimens subjected to AWJM with the jet feed velocity lower than 60 mm/min have a progressive-digressive nature. With increasing the jet feed velocity (above 60 mm/min), the nature of the abrasive material curve becomes less favourable of the digressive/progressive nature. 

**Figure 11:**
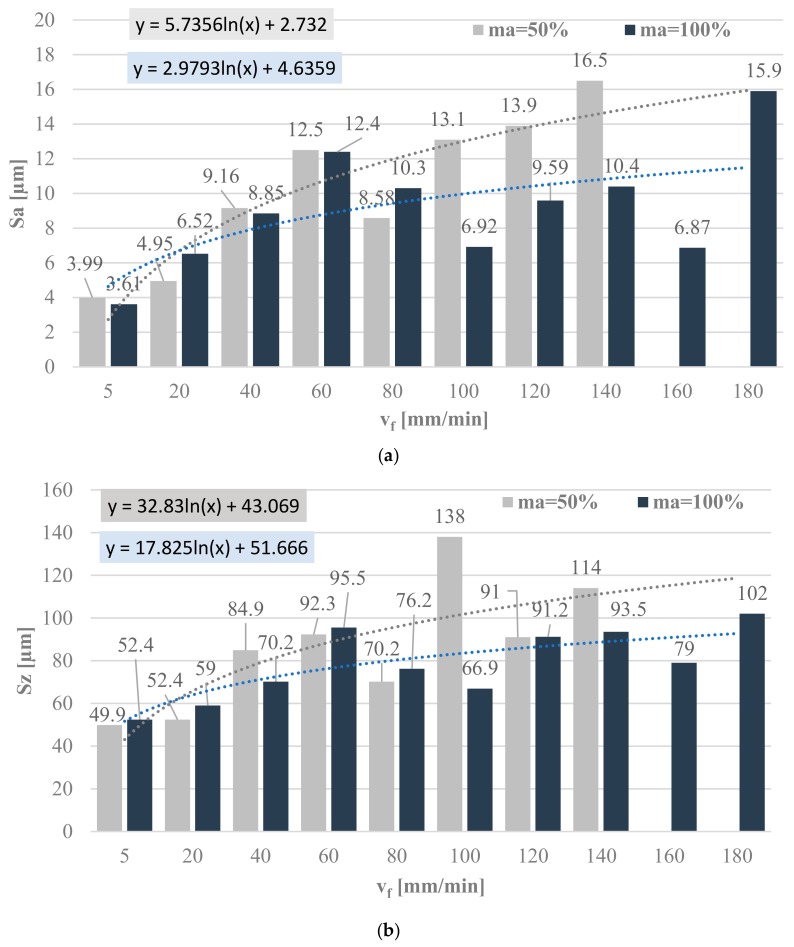
3D area roughness parameters: (**a**) Sa and (**b**) Sz.

Figure 11 shows the 3D area roughness parameters, Sa (Figure 11a) and Sz (Figure 11b), for the abrasive flow rates of 50% and 100% as a function of jet feed velocity. The graphic interpretation of the selected area roughness parameters 3D is shown as in the case of the 2D parameters. 

The results demonstrate that the surface roughness parameter Sa increases as a function of the applied jet feed velocity. For the abrasive flow rate of 50%, the increase in the jet feed velocity to over 140 mm/min made the measurement of surface roughness parameters impossible. In the case of a jet feed velocity range from 5 mm/min to 80 mm/min, no significant changes can be observed in the surface roughness parameter Sa for the abrasive flow rates of 50% and 100%. When the jet feed velocity exceeds 100 mm/min, there is a significant increase in the value of Sa for the abrasive flow rate of 50% when compared to that obtained for the 100% flow rate. 

Similarly to the surface roughness parameter Sa, Sz increases along with increasing the jet feed velocity. The highest increase in the Sz parameter was observed for the jet feed velocity of 100 mm/min during machining with the abrasive flow rate of 50% compared to that obtained in machining with the abrasive flow rate of 100%. This increase amounts to approx. 100%.

### 3.4. Specimen Surface Microhardness 

Microhardness was examined in different regions of the specimen for a single jet feed velocity value (v_f_ = 5 mm/min) considering whether the measurement was possible (no striation marks, irregularities). 

**Figure 12:**
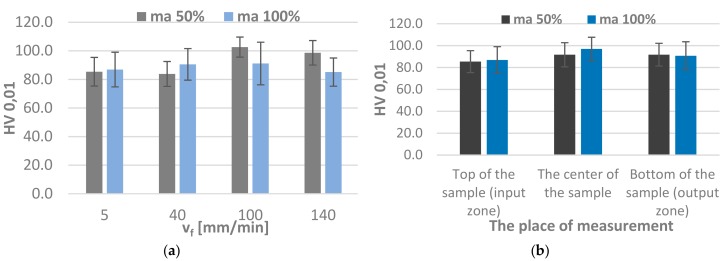
Microhardness: (**a**) when jet feed velocity v_f_ is changed (**b**) in different specimen region when v_f_ = 5 mm/min

As seen in Figure 12b, there are no significant differences in microhardness in different specimen regions. In contrast, the change in the jet feed velocity (Figure 12a) leads to an increase in the average microhardness of the specimens machined with the abrasive flow rate m_a_ = 50%. When m_a_ = 100%, this effect is negligible.

### 3.5. Numerical Modelling of Surface Roughness Parameters by Artificial Neural Networks 

In the simulation of every surface roughness parameter, 100 networks were generated, out of which 3 best-fit networks were selected per every tested roughness parameter, in compliance with the assumption of the research methodology. Their characteristics are given in Table 5.

**Figure 13:**
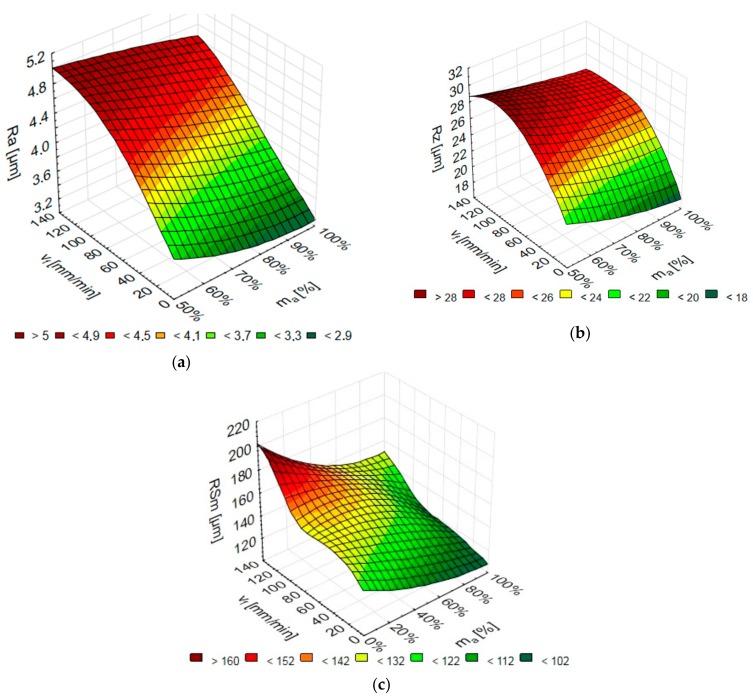
Numerical results of: (**a**) Ra, (**b**) Rz, (**c**) RSm depending on the jet feed velocity v_f_ and abrasive flow rate m_a_ (Ra: MLP 2-8-1, Rz: RBF 2-7-1, RSm: RBF 2-6-1).

The analysis of the obtained models of neural networks reveals that the best surface roughness results were obtained for the following networks: arithmetical mean roughness of the profile Ra for MLP 2-8-1 (Network 2 with eight neurons), maximum height of the profile for Rz: RBF 2-7-1 (Network 5 with seven neurons), while for the mean groove spacing RSm: RBF 2-6-1 Network 5 with six neurons). The numerical results of the surface roughness parameters Ra, Rz, and RSm versus the jet feed velocity v_f_ and the abrasive flow rate m_a_ for the above networks are given in Figure 13. Entering the values of v_f_ and m_a_ into the Statistica programme, we can obtain values of the corresponding surface roughness parameters (Ra, Rz, RSm).

**Figure 14:**
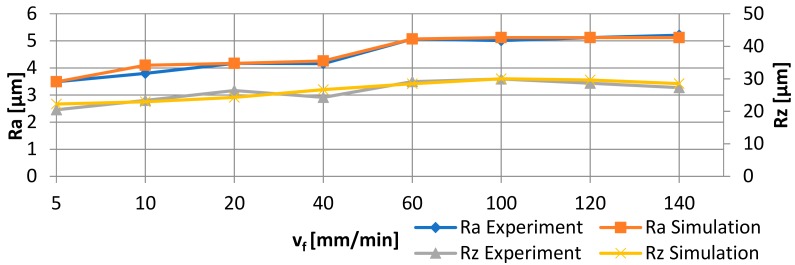
Comparison of experimental and numerical results depending on the jet feed velocity v_f_ for abrasive flow rate m_a_ = 50% and MLP 2-8-1 (Ra and Rz).

To illustrate the accuracy of the modelled networks, Figure 14 shows the comparison of the experimental arithmetic average roughness of the assessed profile Ra and the maximum height of the profile Rz with the numerical values depending on the jet feed velocity v_f_ for the abrasive flow rate m_a_ = 50%. Compared to the real values of the surface roughness parameters, the relative error of Ra is within the range of (0–8)%, that of Rz = (0–9.75)%, whereas the RSm error ranges (0–5)%. 

The numerical results have the acceptable level of error, below 10%. This results from the fact that artificial neural networks provide an effective tool for simulating, e.g., parameters after abrasive water jet machining. With these characteristics in hand, it is possible to elaborate effective numerical models. Artificial neural networks can be effectively used for numerical modelling of machining processes.

## 4. Conclusions

Based on the obtained results and their analysis, the following conclusions can be formulated:

1. The AWJM method can be used to produce semi-finished products whose surface requires no further treatment (for the abrasive flow rate m_a_ = 50%—up to v_f_ = 40 mm/min and for m_a_ = 100%—up to v_f_ = 80 mm/min).
2. For the tested specimen height (approx. 56 mm), it seems justified that the finish machining should be performed to reduce the surface inclination angle (removal of specimen surface chamfering).
3. It can be observed that the investigated 2D roughness parameters show a tendency to increase at the water jet entrance zone (up to approx. 80–100 mm/min), after that their surface roughness parameters become stable (100–140 mm/min), and then there is a further decrease in the investigated 2D surface roughness parameters.
4. In the central region of the specimen, the average values of Ra and Rz increase to the jet feed velocity of v_f_ = 20 mm/min, and then their values become stable.
5. The change in the jet feed velocity v_f_ leads to increasing the average microhardness of the specimens machined with the abrasive flow rate m_a_ = 50%. When m_a_ = 100%, this effect is negligible. No significant differences were observed in the microhardness of different regions of the specimen (when v_f_ = 5 mm/min).
6. It was found that the surfaces created during the abrasive water jet machining of AZ91D specimens for the jet feed velocity of below 60 mm/min are characterized by a progressive/digressive nature of the material shape curve. With increasing the jet feed velocity (above 60 mm/min), the nature of the curve becomes less favourable and changes into digressive/regressive.
7. The results of the experiments show the value of Sa increased by approx. 400% and Sz by 200% in the function of feed velocity.
8. The error margin of obtained numerical results does not exceed 10%, which determines that neural networks are a suitable tool for the modelling of roughness parameters in AWJM depending on the jet feed velocity v_f_ and the abrasive flow rate m_a_.
9. Having established relationships between input and output process parameters (machining parameters and roughness parameters) it is possible to set the machining conditions producing optimal results without the need for any preliminary tests.

## Figures and Tables

**Table 1:** The chemical composition of the workpiece AZ91D alloy.

Chemical Composition (wt %)	Al	Zn	Mn	Si	Cu	Fe	Ni	Be	Mg
AZ91D	8.91	0.66	0.22	0.016	0.002	0.002	0.001	0.001	rest/others

**Table 2:** Operational parameters of the analysed high-pressure abrasive water jet machining process.

Nozzle Diameter	0.7 mm
Abrasive	Garnet 80 mesh
Abrasive flow rate	0.5 kg/min
Stand-off distance	3 mm
Nozzle width	60 mm
Pressure	350 MPa

**Table 3:** Selected isometric images of AZ91D specimen surfaces after AWJM.

v_f_ (mm/min)	Abrasive Material Flow Rate—m_a_	
	50%	100%
5	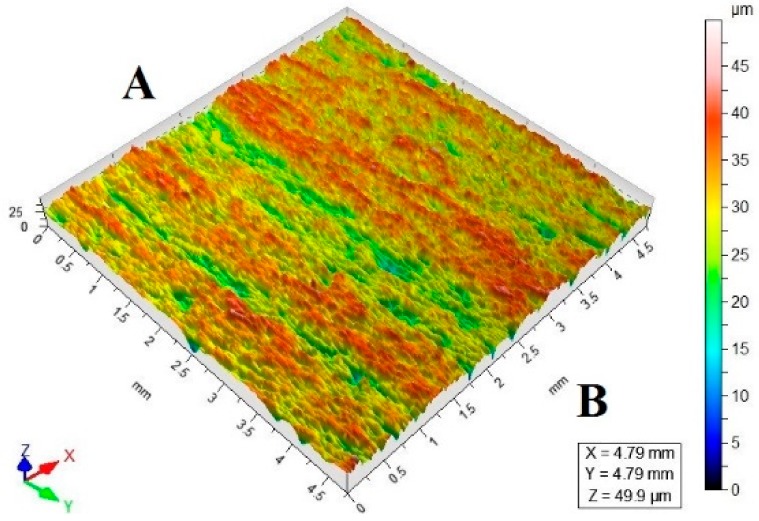	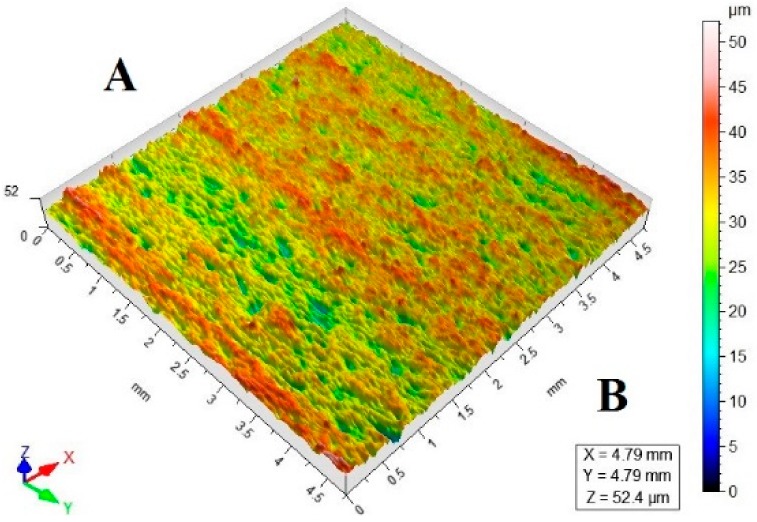
60	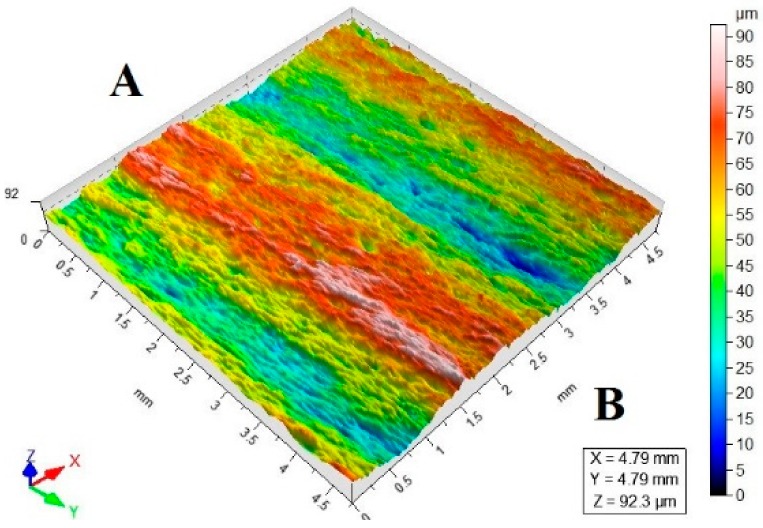	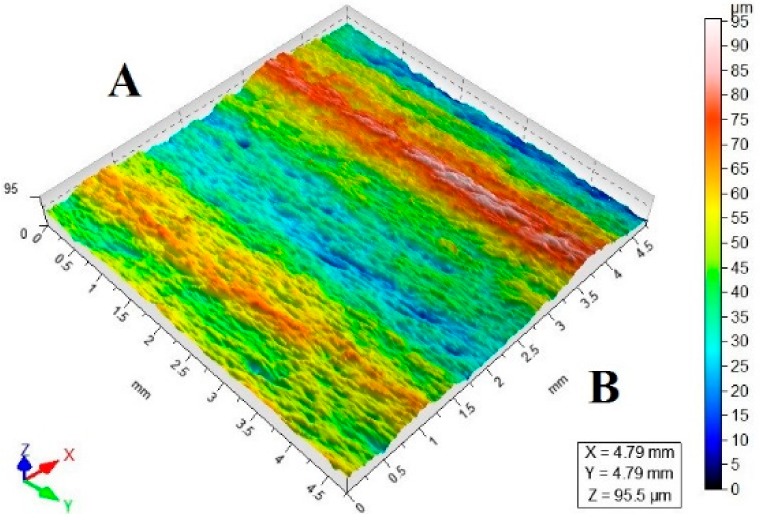
140	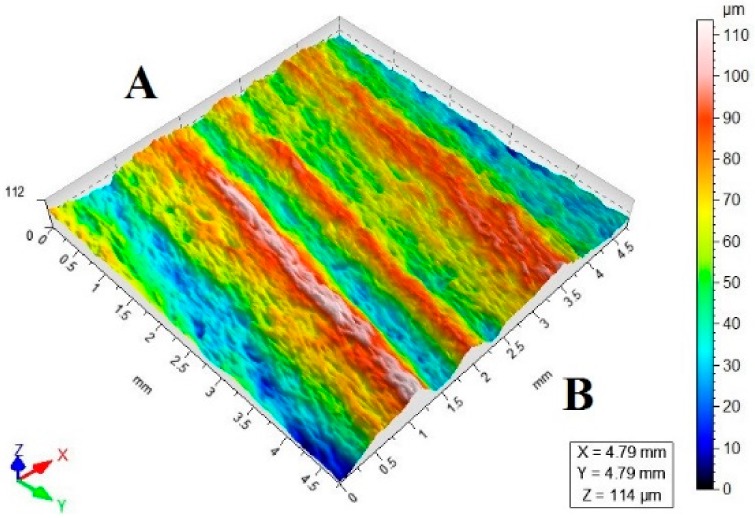	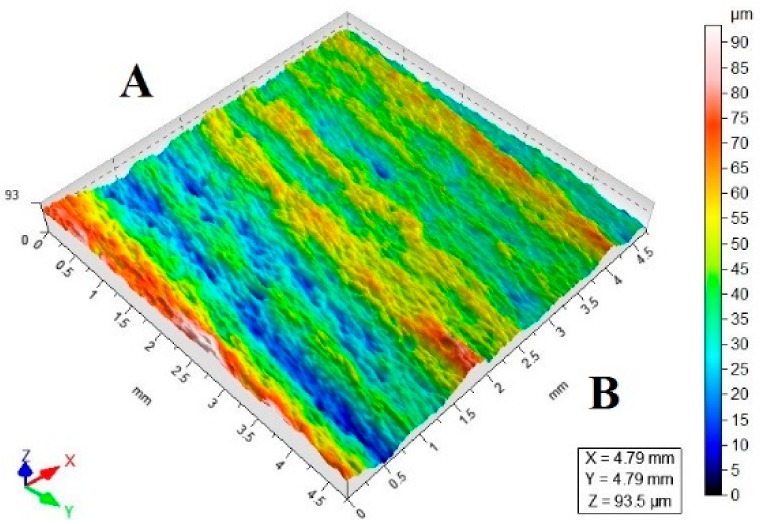

**Table 4:** Abbott-Firestone curve describing abrasive material flow rate.

v_f_ (mm/min)	Abrasive Material Flow Rate—m_a_	
	50%	100%
5	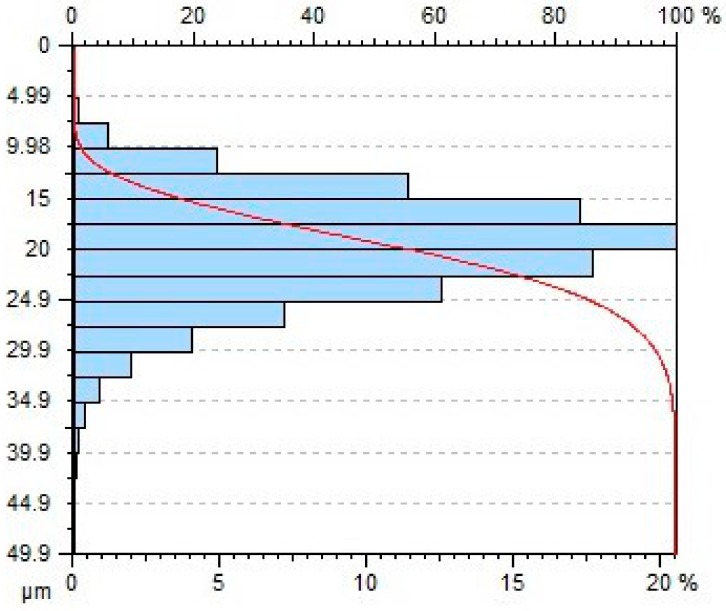	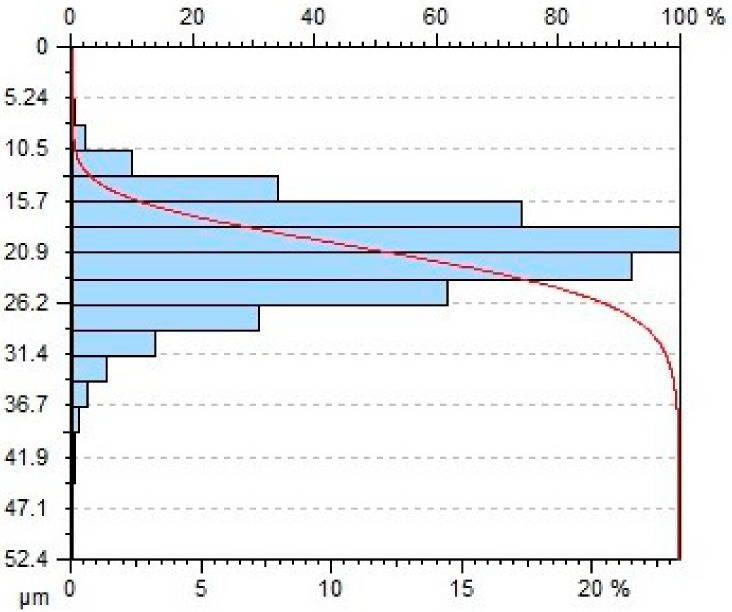
60	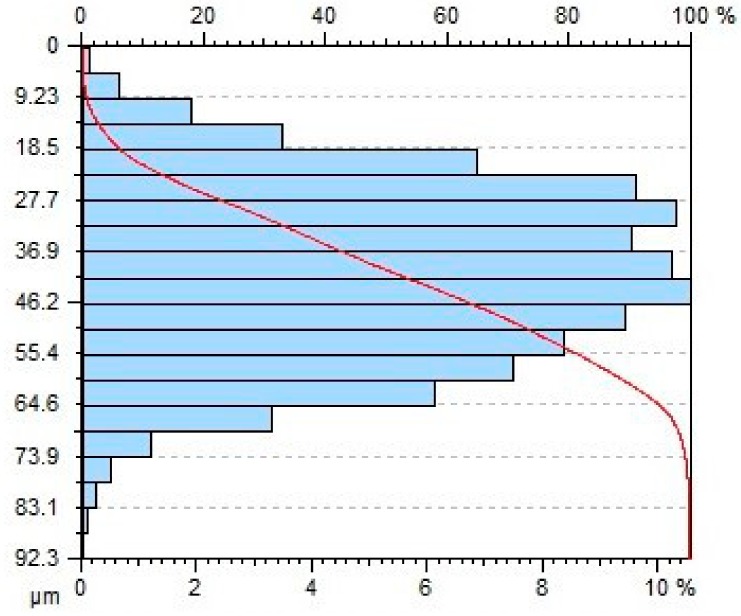	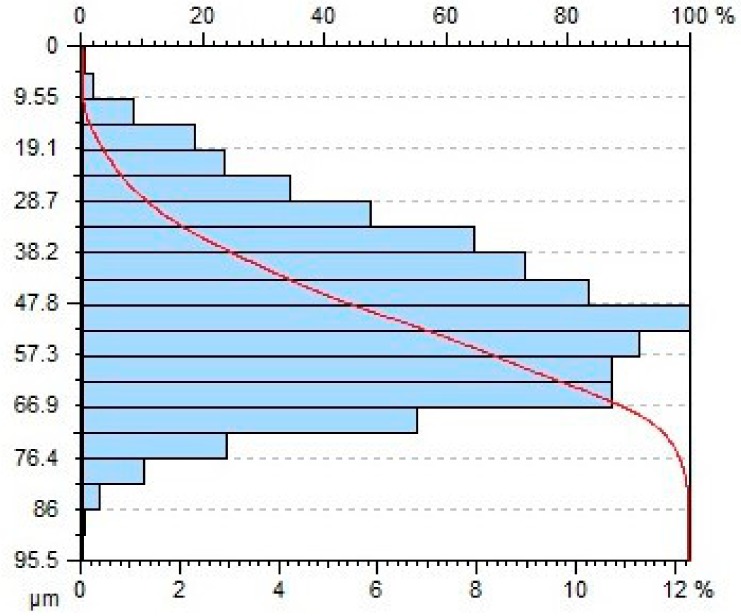
140	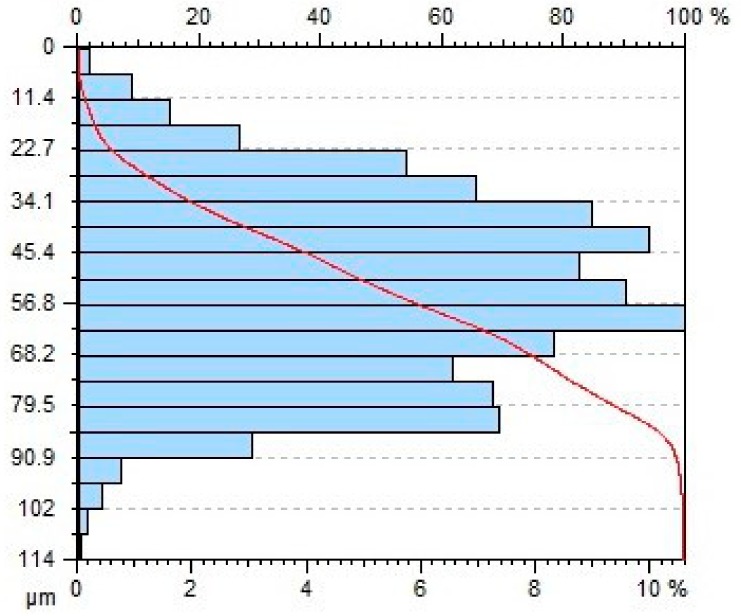	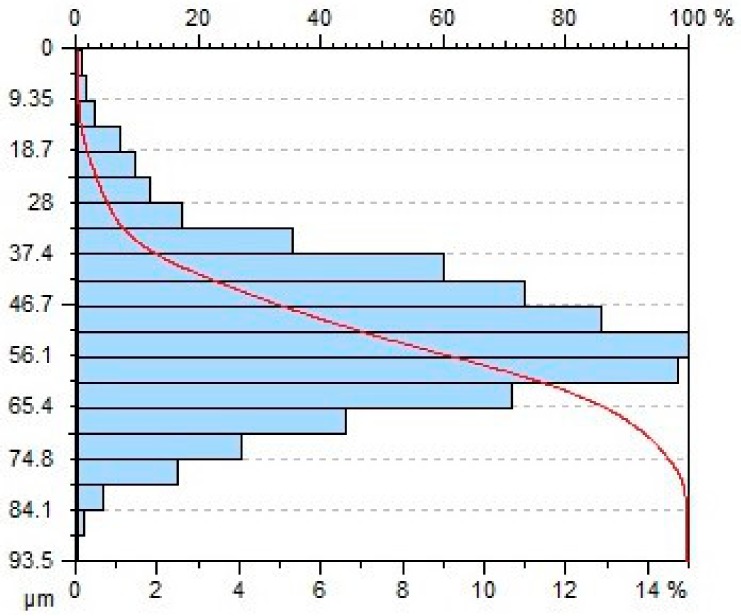

**Table 5:** Characteristics of MLP and RBF networks for surface roughness parameters Ra, Rz, and RSm.

Network No.	Network Name	Quality (Training, %)	Quality (Validation, %)	Error (Training)	Error (Validation)	Activation (Hidden)	Activation (Output)
Arithmetical mean roughness of the profile, Ra							
1	RBF 2-2-1	81.14	98.03	0.086	0.222	Gaussian	Linear
2	MLP 2-8-1	96.88	95.66	0.017	0.026	Sinusoidal	Sinusoidal
3	MLP 2-4-1	93.72	95.55	0.034	0.043	Sinusoidal	Logistic
Maximum height of the profile, Rz							
4	RBF 2-8-1	94.92	90.18	0.687	0.973	Gaussian	Linear
5	RBF 2-7-1	95.94	90.44	0.551	0.916	Gaussian	Linear
6	MLP 2-6-1	93.78	89.21	0.838	0.992	Sinusoidal	Linear
Mean width of profile elements, Rsm							
7	MLP 2-8-1	98.62	99.49	5.053	10.747	Linear	Logistic
8	RBF 2-6-1	98.67	99.62	4.363	5.437	Gaussian	Linear
9	MLP 2-4-1	99.96	98.80	0.119	9.331	Tanh	Exponential
